# Is lifelong endurance training associated with maintaining levels of testosterone, interleukin-10, and body fat in middle-aged males?

**Published:** 2021-07-16

**Authors:** Sara Duarte Gutierrez, Samuel da Silva Aguiar, Lucas Pinheiro Barbosa, Patrick Anderson Santos, Larissa Alves Maciel, Patrício Lopes de Araújo Leite, Thiago dos Santos Rosa, Lysleine Alves de Deus, John Eugene Lewis, Herbert Gustavo Simões

**Affiliations:** ^1^Graduate Program in Physical Education, Catholic University of Brasília, Brasília, Brazil; ^2^Graduate Program in Medicine, Catholic University of Brasilia, Brasilia, Brazil; ^3^Department of Physical Education, University Center UDF, Brasília, Brazil; ^4^Department of Psychiatry and Behavioral Sciences, Miller School of Medicine, University of Miami, Miami, Florida, United States

**Keywords:** aging, chronic training, anti-inflammatory markers, body composition

## Abstract

**Background:**

Aging is associated with a gradual physiological decline, including an imbalance in hormone profile, increased adiposity, and reduced anti-inflammatory cytokines. However, lifelong physical exercise mitigates aging, as observed in endurance-trained middle-aged athletes (EMA).

**Aim:**

We compared and associated testosterone, interleukin 10 (IL-10), and body fat in EMA and untrained age-matched individuals (UAM).

**Methods:**

Participants were EMA (*n*=25; 51.48±9.49 years) and UAM (*n*=23; 46.0±9.37 years). Both groups underwent body composition measurements (evaluated by a skinfold protocol) and blood sampling for IL-10 (assessed through ELISA^®^ kit) and testosterone (assessed with Roche Diagnostics^®^ kit, Mannheim, Germany, by chemiluminescence technique in a third-party laboratory).

**Results:**

EMA had lower body fat (14.15±3.82% vs. 23.42±4.95%; *P*<0.05), higher testosterone (751.68±191.45 ng/dL vs. 493.04±175.15 ng/dL; *P*<0.05), and higher IL-10 (8.00±1.21 pg/mL vs. 5.89±1.16 pg/mL; *P*<0.05) compared to UAM. A significant linear correlation was found between testosterone and IL-10 (r=0.56; *P*=0.001), whereas significant inverse correlations were observed between body fat and testosterone (r=–0.52; *P*=0.001) and body fat and IL-10 (r=–0.69; *P*=0.001).

**Conclusions:**

EMA had higher levels of IL-10 and testosterone and lower body fat in comparison with UAM. In addition, higher IL-10 was associated with increased levels of circulating testosterone and lower body fat.

**Relevance for Patients:**

The adoption of endurance training as part of a healthy lifestyle may contribute to decreasing age-related testosterone reduction, besides reducing markers of inflammaging, preventing the occurrence of chronic age-related diseases, and thus contributing to healthy aging. For people who already have chronic diseases, physical exercise can shift the immune system toward a more anti-inflammatory profile and, thus, improve their pathological condition. In both cases, physical exercise can help attenuate the decline in testosterone, decrease body fat, and increase anti-inflammatory levels.

## 1. Introduction

The aging process is associated with the clinical condition of sarcopenia [1], whose pathophysiology is associated with progressive degeneration of muscle mass and strength, and consequently, a reduction of musculoskeletal function [2]. Sarcopenia is considered one of the major geriatric syndromes that favor the increase in morbidity and mortality of affected individuals [3]. Reduced levels of androgen hormones, especially testosterone, have been frequently observed during aging and have been associated not only with sarcopenia, but also with increased body fat, in parallel with high concentrations of pro-inflammatory cytokines [4,5]. These features may be related to the hypofunction of the hypothalamus-pituitary-gonadal axis and low stimulation of Leydig cells [6,7], probably associated with increased oxidative stress in these cells as we age, which would lead to a decrease in androgen hormone levels, such as testosterone [8]. On the other hand, individuals with a high level of physical fitness, who maintain a balanced diet, lower body fat, and control of stress throughout life, have a lower chronic inflammatory state and higher circulating levels of testosterone [9-11].

Endurance-trained middle-aged athletes (EMA) are individuals over 35 years old, who adhere to consistent and intensive physical training for decades, participating in national and international competitions at a high level. EMA have attenuated aging successfully [12-14] as a result of chronic physiological adaptations, including better body composition [14-16], more favorable oxidative and inflammatory balance [15-17], and maintenance of androgen hormones [4,11,18] compared to untrained age-matched individuals (UAM). In addition, long-term physical exercise also reduces the risk of diseases and disorders, such as metabolic syndrome [19], hypogonadism [20], and neoplasms [21,22].

Testosterone exerts an inhibitory effect on pro-inflammatory cytokines [23], while its low level may be correlated with the expression of inflammatory markers, enhanced by increased body fat [24]. Minuzzi *et al*. [4] demonstrated that master athletes have an interleukin 10 (IL-10) concentration 27% higher compared to middle-aged controls, and their better body composition was significantly associated with an improved immune response. General physiological function is preserved in chronically trained individuals [25,26] that in turn may be associated with the attenuation of age-related decline in circulating testosterone of master athletes [25]. However, the relationships among testosterone concentration, body composition, and pro-inflammatory cytokines, particularly IL-10, have not been investigated in lifelong trained middle-aged individuals. Therefore, we wanted to compare and associate an anti-inflammatory parameter, IL-10, with testosterone and body fat of EMA and UAM. The hypothesis was that EMA have higher testosterone and IL-10 and lower body fat compared to UAM, with testosterone being positively associated with IL-10 and negatively associated with body fat.

## 2. Materials and Methods

This study was approved by the Ethics and Research Committee of the Catholic University of Brasilia (protocol: 3,779,535), and it was conducted according to the Helsinki declaration. All volunteers signed an informed consent form, with all procedures explained in a clear and complete manner.

### 2.1. Participants

A convenience sample of male EMA and UAM was used for the present analysis. These subjects were recruited as part of previous research carried out at the Catholic University of Brasilia. EMA were recruited at national and international athletic competitions and from personal recommendations from other athletes. The inclusion criteria for EMA were: (1) training continuously for at least 15 years; (2) continuing to compete in national and international endurance running events at the time of data collection; (3) not having a diagnosis of recent infection or chronic metabolic diseases; and (4) not taking hormone replacement or any type of pharmaceutical stimulant or depressant of the immune system. The inclusion criteria for UAM were (1) being sedentary; (2) not having a diagnosis of recent infection or chronic metabolic diseases; and (3) not taking hormone replacement or any type of pharmaceutical stimulant or depressant of the immune system.

### 2.2. General procedures

All volunteers arrived at the laboratory in the morning (between 7:00 am and 8:00 am), on days according to the volunteer’s convenience and availability, with 8-h fasting, and abstaining from physical exercise for at least 24 h before procedures. The blood collections of 2 samples of ~4 mL were drawn and deposited each in Vacutainer tubes with and without EDTA. Those procedures were conducted on the same day of anamnesis and anthropometric measurements. All blood samples were collected from an antecubital vein and centrifuged at 1500 turns for 15 min, to determine IL-10 and total testosterone, and stored in cryogenic vials at −80°C. Serum IL-10 was analyzed in triplicate by ELISA^®^ according to the manufacturer’s instructions (R and D Systems, Minneapolis, MN, USA). The detectable limit for IL-10 was 1.0 pg/mL. The overall inter-assay coefficient of variation for IL-10 was 8%. The total testosterone fraction was analyzed in a third-party reference laboratory, with the Atellica^®^ - Siemens^®^ automatic immunoassay equipment, using the chemiluminescence technique [27]. A Roche commercial kit (Roche Diagnostics^®^, Mannheim, Germany) was applied to the Modular E170 automated platform, which uses biotinylated anti-testosterone antibody and a testosterone derivative labeled with a ruthenium complex. The separation was performed with microparticles covered with streptavidin, captured by magnetic action and subjected to washing. After this procedure, the reading was performed by applying a voltage that induced the emission of light by the ruthenium complex (electrochemiluminescent test). Standard solutions were prepared for all procedures, one containing only the reagent and a control solution containing the reagent plus a known solution, in duplicate.

Body mass index (BMI) was calculated from weight in kilograms divided by height in meters squared (kg/m^2^). The relative body fat was estimated using the seven skinfolds protocol proposed by Jackson and Pollock, obtaining the values of the pectoral, axillary, triceps, subscapular, medial thigh, suprailliac, and abdominal folds [28]. A single researcher measured all skinfolds with a Lange^®^ caliper (Cambridge Scientific Instruments, MA, USA). Body density was then calculated following the equation: Body density (g/cm^3^)=1.112–0.00043499* (sum of 7 skinfolds)+0.00000055* (sum of 7 skinfolds) 2–0.00028826* (Age), and converted into a percentage of body fat, using a formula: Fat%=([4.95/DENS]–4.50)×100 [29].

### 2.3 Statistical analysis

The normality of the data was verified with the Shapiro-Wilk test. The data were expressed as mean±standard deviation. Independent samples *t*-tests were conducted to compare each variable between the EMA and UAM groups. In addition, the effect size (Cohen’s d) was calculated [30]. Pearson’s correlation coefficients were calculated to determine the relationships among testosterone, IL-10, and body fat, adjusted for BMI and age together. The level of significance was set at *P*<0.05. The total sample size in this study conferred statistical power of 86% (*post hoc*) with a significance level of a=0.05 and a large effect size of d=0.8. All procedures were performed using GraphPad Prism (v. 6.0), Gpower^®^ (v. 3.1), and SPSS 21.

## 3. Results

The sample consisted of 25 EMA (51.48±9.49 years of age; 21.71±10.19 years of training; 102.08±39 min of daily training; 4.67±1.4 days of weekly aerobic training; and 2.23±1.22 days of weekly strength training) and 23 UAM (46.0±9.37 years). Compared to the EMA, the UAM group had higher body weight (+28.3%; *P*=0.001), BMI (+22.8%, *P*=0.001), and height (+2.25%, *P*=0.047). Age differed slightly between groups (*P*=0.056). The age range of EMA was 38–71 years (52.21±8.959 years). The age range of UAM was 36–65 years (48.22±8.135 years). The age range of the two groups together was 36-71 years (50.50±8.744 years). The sample characteristics are shown in Table 1.

**Table 1:** Biometric characteristics of UAM and EMA

Variables	UAM	EMA	*P*-value
Age (years)	46.0±9.37	51.48±9.49	0.056
Weight (kg)	91.1±15.29	70.99±7.35	0.001
Height (m)	1.78±0.06	1.74±0.06	0.047
BMI (kg-m^2^)	28.48±4.47	23.19±2.09	0.001

Data expressed as mean and (±) standard deviation. UAM, untrained age-matched individuals; EMA, endurance-trained middle-aged athletes.

**Figure 1:**
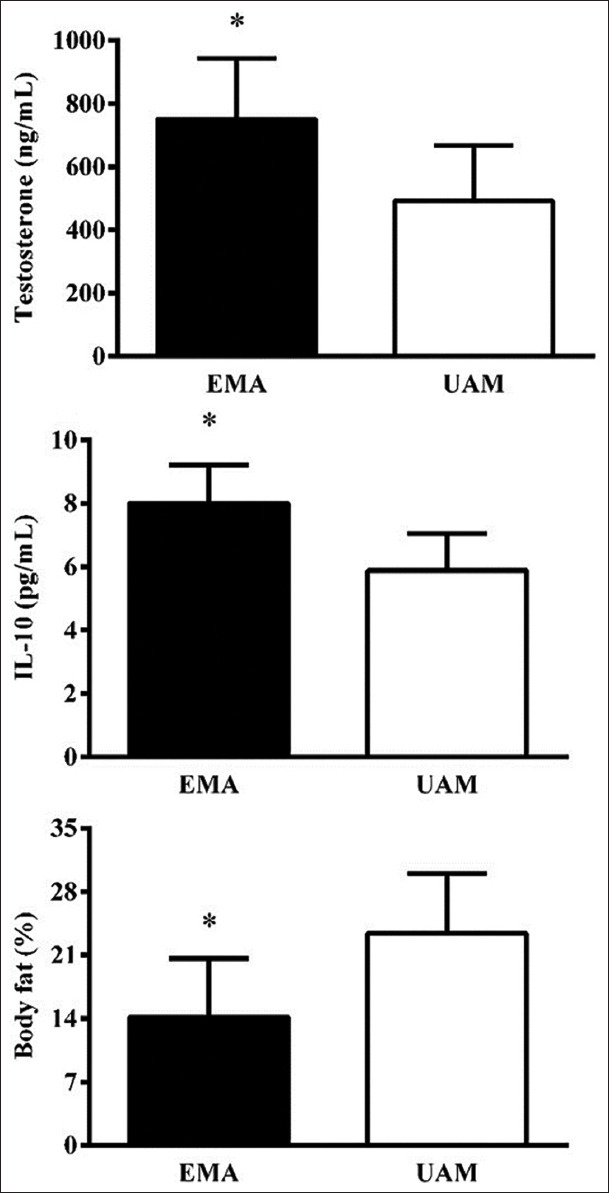
Testosterone, IL-10, and body fat of endurance-trained middle-aged and untrained age-matched individuals. IL-10, interleukin 10; * statistical difference; *P*=0.001.

Testosterone was higher (*P*=0.001) in EMA (751.68±191.45 ng/dL) compared to UAM (493.04±175.15 ng/dL), with a Cohen’s d of 1.41, indicating a very large effect size. IL-10 was higher (*P*=0.001) for EMA (8.00±1.21 pg/mL) compared to UAM (5.89±1.16 pg/dL), with a very large effect size (d=1.78). Body fat was lower (*P*=0.001) in EMA (14.15±3.82%) compared to UAM (23.42±4.95%), with a very large effect size (d=2.10); (Figure 1).

**Figure 2:**
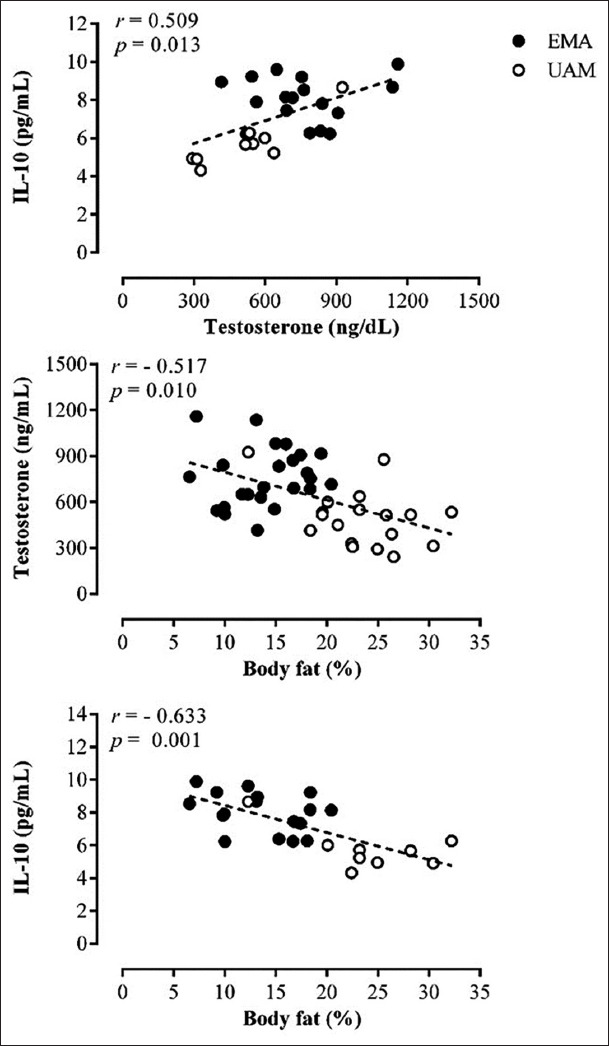
Relationship between IL-10 and testosterone (a), testosterone and body fat (b), and IL-10 and body fat (c) of resistance-trained and untrained middle-aged individuals, adjusted for body mass index and age together. IL-10, interleukin 10.

A significant linear correlation was found between testosterone and IL-10 (r=0.56; *P*=0.001). In addition, body fat was inversely correlated with testosterone (r=–0.52; *P*=0.001) and IL-10 (r=–0.69; *P*=0.001); (Figure 2).

## 4. Discussion

In the present study, IL-10 and testosterone were compared and correlated in EMA and UAM. EMA were shown to have increased IL-10, whereas it was lower in UAM, possibly suggesting an initial deregulation of inflammatory status for untrained middle aged men [6,31]. In addition, EMA had a higher testosterone concentration compared to UAM. Moreover, a positive correlation between testosterone and IL-10 and a negative correlation between testosterone and body fat demonstrated that EMA are aging healthily [32].

Aging is generally associated with a systemic functional decline in health, due to an accumulation of genetic mutations and cellular damage throughout life [33]. However, exercise and other healthy habits modulate several biomarkers of aging [4,14-17,34], and EMA are a successful model of adequate nutrition, stress management, and consistent training for decades [13]. This lifestyle seems to decrease inflammation [4,17,16,34] and mitigate the decline of testosterone during the usual aging process [35], which is corroborated by the findings of the present study.

Among men, testicular hypofunction occurs over the lifespan due to an increase in reactive oxygen species (ROS) and a decrease in antioxidant defenses, which reduce the sensitivity of luteinizing hormone (LH) receptors and, consequently, decrease the biosynthesis and secretion of testosterone [8]. Further, low testosterone is a predictor of the development of type 2 diabetes, hypertriglyceridemia, hypercholesterolemia, and cardiovascular diseases [36]. Freeman *et al*. [37] demonstrated that androgen deficiency is a pro-inflammatory modulator, and these cytokines contribute to dysfunctional vascular remodeling from endothelial inflammation. Thus, low testosterone is typically associated with functional decline and a predisposition to chronic diseases mediated by inflammation, mainly cardiovascular diseases, whose morbidity and mortality increase proportionally with age [38].

In addition, we showed that UAM have lower IL-10, an anti-inflammatory marker, and decreased testosterone, while these were elevated in EMA. While testosterone levels were positively associated with IL-10, body fat was negatively associated with both testosterone and IL-10. Besides being the first study to date to demonstrate the relationships among testosterone, IL-10, and body composition in endurance trained middle aged individuals, our findings reinforce that chronic exercise training and the maintenance of low body fat may be pivotal for a healthy anti-inflammatory status and testosterone levels as previously demonstrated [35,39]. Moreover, lifelong exercise training also improves antioxidant defenses [8,17,15,16], and all of the above-mentioned aspects are associated with longevity and healthy aging [13].

According to Bianchi [23], testosterone is immunoregulatory by suppressing the synthesis and proliferation of pro-inflammatory cytokines, such as TNF-alpha, thereby reducing the risk of obesity, atherosclerosis, and metabolic syndrome. Furthermore, Bini [40] demonstrated that an increase in pro-inflammatory cytokines and a decrease in anti-inflammatory cytokines during infectious-inflammatory conditions stimulate excess production of ROS. While exercise may initially protect against pathogens, over time chronic inflammation may lower antioxidant defenses, leading to Leydig cell hypofunction and gonadal resistance to LH stimulation, interfering in steroidogenesis and biosynthesis for the production of testicular testosterone [8]. Thus, higher testosterone in the EMA group may at least partially explain their elevated IL-10, demonstrating positive and continuous feedback between the immune and endocrine systems.

The possible limitations of this study include not measuring nutritional status and dietary intake and not controlling for the use of dietary supplementation among participants. Furthermore, the level of physical fitness of participants was not analyzed because of the short time researchers had with each participant and also because of limitations inherent to international trips for data collection. The initial intent of the study was to form two groups only, athletes and untrained individuals, regardless of their level of physical activity. However, it is important to mention that master athletes have better nutrition and vigorous training routines than non-athletes. In addition, we studied a group of high-level master endurance athletes, which is a very specific and small portion of the population.

## 5. Conclusions

Endurance trained middle-aged participants had higher testosterone and IL-10 concentrations and lower body fat compared to untrained peers. Testosterone concentrations were associated with a better anti-inflammatory profile and improved body composition. Our athlete sample was comprised of people whose training regimen may result in attenuated inflammaging without chronic disease. Given the global preponderance of chronic diseases, a more widespread adoption of endurance training as part of an overall healthy lifestyle should eventually reduce the occurrence of inflammaging and chronic diseases in the population. Proper physical training may have clinical applications for attenuating testosterone decline, decreasing body fat, and maintaining an adequate anti-inflammatory status, especially in middle age. Longitudinal studies with more subjects and involving master athletes from other sports, such as sprinting, are needed in addition to the current findings to determine how testosterone, IL-10, and body fat are related to functional and health status over time, and the influence of exercise training mode.
